# Embedding sustainable physical activities into the everyday lives of adults with intellectual disabilities: a randomised controlled trial

**DOI:** 10.1186/1471-2458-14-1038

**Published:** 2014-10-05

**Authors:** Kerrie Lante, Roger J Stancliffe, Adrian Bauman, Hidde P van der Ploeg, Stephen Jan, Glen M Davis

**Affiliations:** Faculty of Medicine, Nursing and Health Sciences, Flinders University, Adelaide, SA 5001 Australia; Centre for Disability Research and Policy, The University of Sydney, PO Box 170, Lidcombe, NSW 1825 Australia; Sydney School of Public Health, The University of Sydney, Sydney, NSW 2006 Australia; Department of Public and Occupational Health, VU University Medical Center Amsterdam, Amsterdam, The Netherlands; The George Institute for Global Health, Camperdown, NSW 2050 Australia; Discipline of Exercise and Sports Science, The University of Sydney, Lidcombe, NSW 1825 Australia

**Keywords:** Intellectual disability, Community living, Physical activity, Exercise

## Abstract

**Background:**

Adults with intellectual disability (ID) are physically very inactive. This study will compare two approaches to increasing physical activity in adults with ID: a lifestyle physical activity (light-moderate intensity) approach and a structured exercise (moderate-vigorous intensity) approach. The trial will compare the short-term (3-month) and long-term (9-month) outcomes and sustainability of each approach with a usual-care control group.

**Methods/Design:**

A three-arm randomised controlled trial (RCT) will be conducted. Ninety adults with ID aged 18-55 will be randomly assigned to one of three groups: 1) a lifestyle physical activity group (*n* = 30), 2) a structured exercise group (*n* = 30), or 3) a usual care control group (*n* = 30). Participants in both groups will receive a 12-week intervention delivered by exercise specialists in the community with disability service staff, after which intervention will continue for 6 months, delivered by disability service staff only. Primary outcomes are aerobic fitness, 12-hour energy expenditure, and proxy-reported everyday physical activity. Secondary outcomes include objectively assessed physical activity and sedentary behaviour, intervention compliance, functional walking capacity, participation in domestic activities, muscle strength, body composition, psychosocial outcomes, quality of life and health care costs.

**Discussion:**

The trial results will determine the effectiveness and sustainability of two approaches to increasing physical activity and exercise among adults with ID.

**Trial registration:**

ISRCTN77889248 (18 April 2012).

## Background

Adults with intellectual disability (ID) are substantially less physically active than the general community [1–3] which may contribute to preventable physical and mental health problems [4, 5]. In Australia, the proportion of adults with ID who meet national guidelines for physical activity [6] is only around half that of the general community [7]. People with ID have a right to good health and a healthy lifestyle, yet without adequate exercise and physical activity they are more likely to experience a lower quality of life and a higher level of physical and mental ill health. Failing to address these issues places an increased burden on families and carers, as well as on disability services and health systems. Such preventable health problems can increase dependency (requiring higher disability service staffing levels), add to absenteeism from disability employment, and result in premature withdrawal from the workforce and greater health care utilisation [5].

The majority of adults with ID have the potential to meet the physical activity guidelines [8–10], but people with ID need direct personal support to plan, organise, travel to and participate in physical activities. There is a ready source of such support: disability service staff who already provide daily (usually 24-hour) support to their clients. However, these staff do not usually have the skills to support physical activity and exercise of appropriate intensity and duration. Crucially, research on ‘Active Support’ from staff has shown (a) that appropriate personal support strongly increases participation in functional everyday activities by adults with ID and (b) that disability staff can be trained to provide it [11, 12].

Physical activity is an important national policy issue in Australia [13]. The objective of this project is to improve the physical activity, aerobic fitness, strength and well-being of adults with ID by increasing their everyday physical activity and exercise levels in a manner that is sustainable in the long-term. This will involve comparing two approaches to disability staff support of physical activity by adults with ID: (I) a lifestyle physical activity (light-moderate intensity [LMI] lifestyle) approach and (II) a structured exercise (moderate-vigorous intensity [MVI] exercise) approach. The effectiveness of the two strategies for increasing physical activity and exercise in adults with ID will be evaluated and compared to a usual-care control group. By contrasting two different approaches to increased physical activity we will compare the short-term (3-month) and long-term (9-month) effectiveness and sustainability of both strategies. In addition, the project will evaluate the cost-effectiveness of both strategies.

## Methods

The trial has been registered on International Standard Randomised Controlled Trial Number Register (ISRCTN77889248).

### Funding

The study is being funded by National Health and Medical Research Council (Australia) Partnership Grant APP1012692, and two disability industry partners, Lorna Hodgkinson Sunshine Home, and House With No Steps.

### Design

A three arm randomised controlled clinical trial (RCT) will be conducted. Exercise specialists will deliver either a 3-month LMI-Lifestyle physical activity program or a structured MVI-Exercise program. During program delivery, exercise specialists will train disability service provider staff so that they can continue the program post intervention. The trial will be conducted with the two partner organisations, which provide services to adults with ID across Sydney, New South Wales, Australia. These services include (a) staff supported accommodation in small group homes or other community-based settings and (b) sheltered employment.

Ethics approval has been obtained from Sydney University Human Research Ethics Committee (HREC 05-2011/13821). Participants and where relevant their caregiver or guardian will be provided with information sheets, and written informed consent will be obtained prior to inclusion in the study and baseline assessment. Any adverse events related to the trial will be recorded and reported to the Human Research Ethics Committee.

### Participants

Ninety adults with ID will be recruited through the disability partner organisations on a rolling schedule. Those interested in participating will be asked to complete a Physical Activity Readiness Questionnaire (PAR-Q) [14] and where concerns are identified by the PAR-Q, medical clearance will need to be obtained in order to participate in the study.

### Inclusion criteria

Participants will be included if:

1. they have intellectual disability
2. they are aged between 18-55 years
3. it is reported they have been physically inactive for the past 12 months or longer
4. they reside within a 1.5-hour commute to the testing site
5. they are able to participate in assessments such as the 6-minute walk test
6. there is a signed informed consent in accordance with the ethics requirements.

### Exclusion criteria

Participants will be excluded if:

1. they have contraindications to participating in exercise programs or outcome assessments, as advised by the primary care physician and/or ineligibility according to American College of Sports Medicine (ACSM) criteria [15].
2. they are judged at risk of self-harm by the disability service provider care staff.

#### Randomization

Participants will be randomly assigned to one of three groups: 1) a LMI-Lifestyle intervention physical activity group 2) a structured MVI-Exercise group or 3) a usual care control group.

#### Intervention

##### Intervention group 1; lifestyle (LMI) physical activity

Exercise specialists from the University of Sydney will use the ‘Active Support’ approach [12] to promote the LMI-Lifestyle intervention. ‘Active Support’ is an evidence-based approach for the long-term support of activities of daily living (e.g., cooking), with training of disability service provider staff to provide tailored support as a central feature. Using the principles underlying active support participants in the LMI-Lifestyle group will be encouraged to engage in a total of 150 minutes per week of LMI physical activity. Participants will be supported for 1 hour (60 minutes) a week by the exercise specialist to engage in individually tailored LMI-Lifestyle activities that are enjoyable (e.g., X-Box Kinnect, swimming, television advertisement exercises) or serve a functional purpose (e.g., walking for transport, hanging out washing). The remaining 90 minutes of weekly physical activity will be supervised by disability staff, who will be encouraged to follow an activity program set out by the university exercise specialist. During the weekly session with the exercise specialist, participants will wear a multi-sensor armband, Sensewear [16] to assess exercise intensity and compliance. Disability service provider staff will participate in the planning, and where appropriate the activity during and beyond the 12-week intervention period.

##### Intervention group 2; structured MVI-exercise

Moderate-vigorous intensity exercise classes led by the university exercise specialists will take place over 12 weeks. Three to six participants will be recruited for each cohort. A MVI-Exercise session will comprise 30 to 45 minutes of cardiovascular exercise and 15 to 20 minutes of muscular strength. Sessions will be conducted three times per week. Exercise intensity will be gradually increased over the 12 week intervention period from 40%-50% of heart rate reserve to 50%-70% of heart rate reserve [17]. Exercise modes will be conducted indoors or outdoors in local community settings (partner organisation’s facilities, parks, community gyms) using a small group approach involving activities like brisk walking, jogging and calisthenics with emphasis upon enjoyable body movements. To assess exercise intensity and compliance participants will wear a multi-sensor armband, Sensewear [16], for estimation of energy expenditure, throughout the initial 12-week intervention. Participants will also wear heart rate monitors (Polar, Port Washington, NY, USA) to ensure that they are exercising in the appropriate target heart rate zone. Partner Organisation staff will participate in the exercise sessions alongside the exercise specialists and will be trained to continue these classes beyond the 12-week intervention period.

##### Intervention group 3; control participants

Participants in the control group will continue with their everyday activities. They will not be involved in any physical activity interventions led by the exercise specialists.

### Outcome assessments

All outcomes will be assessed at baseline, 3-months post-baseline and at 9-months post-baseline. Participants and assessors will be un-blinded. The primary outcome measures are aerobic fitness, energy expenditure and physical activity. Secondary outcomes include intervention compliance, participation in domestic activity, objectively assessed physical activity and sedentary time, muscle strength, functional walking capacity, health care utilisation and cost, quality of life, body composition and psychosocial measures.

### Primary outcome measures

#### Aerobic fitness

To assess aerobic fitness a submaximal exercise aerobic fitness test will be performed. Participants will undertake up to 4 × 5-min leg cycling using an isokinetic cycle ergometer, with resistance adjusted to elicit target heart rates equivalent to 45%, 55%, 65% and 75% of age-adjusted peak heart rate. Using ACSM metabolic equations [18], power output (W) and measured heart rate at each submaximal stage will be used to estimate submaximal oxygen consumption and VO_2_peak (ml•kg•min^-1^).

#### Energy expenditure

To quantify possible change of energy expenditure after the two exercise interventions, 12-hour energy expenditure will be assessed. At baseline, 3-month and 9-month post assessment participants will, on two days, wear a multi-sensor armband, Sensewear [16]. This small, lightweight self-contained device accurately measures and stores energy expenditure and physical activity data in the field.

#### Physical activity

The long, telephone version of the *International Physical Activity Questionnaire* (IPAQ) was adapted to measure the physical activity of adults with ID using proxy respondents [19]. To quantify possible change of physical activity via a simple recall questionnaire, the adapted version of the IPAQ, the IPAQ-ID, will be administered to carers of the adult with ID.

### Secondary outcome measures

#### Objectively assessed physical activity and sedentary time

Participants will wear an Actigraph GT1M/GT3X accelerometer (Actigraph, Pensacola, FL, US) on the right hip attached to an elastic belt, for 7 consecutive days. The Actigraph is a non-invasive device the size and weight of a matchbox that records activity counts and steps taken. Participants wear the accelerometer during waking hours except during water activities. The accelerometer data allows for checks of wearing compliance and can be used to estimate time spent in sedentary, light, moderate, and vigorous physical activities.

#### Intervention compliance

Compliance with physical activities in both intervention groups will be measured through a daily physical activity log. For participants in either intervention group the disability service provider staff will be asked to log each person’s participation in physical activities (type and duration).

#### Functional walking capacity

Participant’s functional walking capacity will be assessed through a 6-Minute Walk Test (6MWT). Participants will be asked to walk as fast as they can for 6 minutes and if required, the participant may briefly stop. The test will be performed indoors, along a flat, hard-surfaced passageway. The total distance walked will be measured and heart rate will be recorded using a heart rate monitor (Polar, Port Washington, NY, USA). This is a well validated test for a range of populations [20].

#### Participation in domestic activity

The 13-item Index of Participation in Domestic Life (IPDL) [21] scale will be administered to carers of the adult with ID. It measures participation in functional domestic activity, not physical activity.

#### Muscle strength

Isometric assessment of participants’ muscle strength will be via dynamometry and include maximal handgrip strength, biceps flexion, triceps extension and knee extension strength [22].

#### Body composition and body fat

The Innerscan Body Composition Monitor (Tanita BC-541, Tsimshatsui East, Kowloon, Hong Kong) will be used by a trained research assistant to measure the participant’s body mass and estimated percentage body fat. Height will also be measured. From the recorded information Body Mass Index (BMI) will be calculated.

#### Psychosocial measures

We will examine the effects of the interventions on attitudes and psychosocial outcomes through self-report and/or proxy interview data. Attitudes towards physical activity (self-efficacy, social support and exercise expectations) will be assessed using the following scales:

##### Self-Efficacy for Activity for persons with Intellectual Disabilities (SE-AID)

This scale [10] is a 6-item self-report scale with internal consistency (alpha) = 0.73, and test-retest reliability intraclass correlation (ICC) = 0.49.

##### Social Support for Activity for persons with Intellectual Disabilities (SS-AID)

This scale [10] has versions for closely related family and staff. The family version is a 7-item self-report scale with internal consistency (alpha) of 0.73, and test-retest reliability (ICC) of 0.79. The staff version has 6 items, with alpha = 0.74 and test-retest ICC = 0.78. The version of the scale administered will be dependent on the participants living circumstances.

##### The exercise perceptions scale

This scale [23] measures expected outcomes of exercise. It is a 9-item self-report scale (alpha = 0.81), and test-retest r = 0.72 [24].

Psychosocial outcomes (depression and health-related quality of life) will be assessed using the following scales:

##### The Glasgow Depression Scale (GDS)

The GDS [25] has self-report and proxy-report versions which correlate strongly (*r* = 0.93). It is specifically designed for people with ID and has excellent internal and test–retest reliability and criterion validity, with 96% specificity and 90% sensitivity. Both versions will be completed, with the proxy-report version being administered to the carers of the adult with ID.

#### Quality of life

##### The Health Utilities Index 2 (HUI 2)

This scale [26] used for economic evaluation will assess quality of life.

#### Health care costs

An economic evaluation will determine the cost-effectiveness of the intervention. To assess the impact on health economics carers of the adult with ID will be given a prospective diary to complete about the person’s use of health services. The diary will ask for information on the number of hospitalizations, any medical consultations (and other medical services) and any medications taken over the period.

The Health Utilities Index 2 (HUI 2) [26] is a widely used generic measure of health state utility used for economic evaluation. The scale will be administered to carers of the adult with ID.

### Statistical analyses

#### Sample size

We will power the study based on the primary outcome of aerobic fitness (VO_2_peak; ml•kg•min^-1^). Our MVI-Exercise approach is similar to a previous study that used a 12-week gym-based intervention for people with ID [27]. They reported substantially better post-test aerobic fitness outcomes (assessed by VO_2_peak) for the intervention group relative to controls (large effect size, Cohen’s d = 0.91). Assuming an effect size of d = 0.80 (equivalent to f =0.40), and using a design with three groups and three repeated measures, power calculations with alpha = 0.05, power = 0.95, and a correlation among repeated measures of 0.5 show that this would require a total sample size of 69 (i.e., 23 per group), to analyse between-group differences using repeated measures ANOVA. Our assumptions are conservative so as to avoid under-powering the study. Therefore, we will recruit 30 participants per group for a total sample of 90, which allows for 23% attrition without compromising power.

#### Analyses

Repeated-measures ANOVA will be used to compare both the intervention and control group participants on primary and secondary outcome measures, at the 3-month and 9-month assessments. To identify personal characteristics of participants and features of their environment that are associated with better outcomes multiple regression will be used. Significance will be set to *p* < 0.05. Analysis will be on the basis of intention to treat, with a secondary per-protocol analysis.

For the economic evaluation a trial based cost-effectiveness analysis, taking a health system perspective, will compare the two exercise interventions with one another and, separately, with usual care (i.e., our control group). The economic evaluation will estimate the incremental cost-effectiveness of each of the interventions in terms of cost per quality adjusted life year gained. Participant level costs associated with use of health services and medications will be derived from proxy-reported utilisation at each follow-up and costed on the basis of standard published costs (e.g., Australian Refined Diagnosis Related Groups cost weights for hospitalisation and Medicare Benefits Schedule and Pharmaceutical Benefits Scheme [PBS] rates for medical services and prescribed medications respectively). Quality of life will be assessed by the HUI 2 via proxy and converted into health state utilities via the UK scoring system [28].

## Discussion

This trial will enable evaluation of the effectiveness of two forms of exercise intervention for adults with ID, an under-researched group in preventive health research. The project has potentially important outcomes for both people with ID and the disability industry.

Through support for increased physical activity and exercise, we will determine if people with ID experience changes in primary outcomes of fitness, energy expenditure and physical activity, as well as a range of important secondary outcomes. We will evaluate outcomes after three months of intervention delivered by exercise specialists and disability service provider staff, and again six months later following continued intervention supported by disability service provider staff alone.

If successful, the project will form the basis for increased disability industry capacity to support healthy behaviours by training disability service provider staff to support physically active lifestyles. For widespread clinical implementation of our approach in the ID sector to be successful, it is necessary (in addition to staff training) to use a simple, low cost method to monitor the effectiveness of the physical activity interventions. One of our primary outcomes, the IPAQ-ID [19], is a low-cost, low-tech tool that disability service staff can be trained to use in everyday clinical practice. Therefore, an additional important outcome of this project will be further evidence to complement initial validation studies [29] of the IPAQ-ID’s capacity to detect important changes in physical activity in this population. This will enable easier future uptake and evaluation of physical activity interventions by the disability industry.

It is expected that this trial will require three years to complete.

### Trial status

Recruitment and intervention will be conducted in four overlapping waves.

## Abbreviations

ID: Intellectual disability
RCT: Randomised controlled trial
LMI: Light-moderate intensity
MVI: Moderate-vigorous intensity
PAR-Q: Physical activity readiness questionnaire.
